# Resting-State Functional Network Scale Effects and Statistical Significance-Based Feature Selection in Machine Learning Classification

**DOI:** 10.1155/2019/9108108

**Published:** 2019-11-04

**Authors:** Hao Guo, Yao Li, Godfred Kim Mensah, Yong Xu, Junjie Chen, Jie Xiang, Dongwei Chen

**Affiliations:** ^1^College of Information and Computer, Taiyuan University of Technology, Taiyuan, China; ^2^Department of Psychiatry, First Hospital of Shanxi Medical University, Taiyuan, China; ^3^School of Electronic Information Engineering, University of Electronic Science and Technology of China, Zhongshan Institute, Zhongshan, China

## Abstract

In recent years, functional brain network topological features have been widely used as classification features. Previous studies have found that network node scale differences caused by different network parcellation definitions significantly affect the structure of the constructed network and its topological properties. However, we still do not know how network scale differences affect the classification accuracy, performance of classification features, and effectiveness of the feature selection strategy using *P* values in terms of the machine learning method. This study used five scale parcellations, involving 90, 256, 497, 1003, and 1501 nodes. Three local properties of resting-state functional brain networks were selected (degree, betweenness centrality, and nodal efficiency), and the support vector machine method was used to construct classifiers to identify patients with major depressive disorder. We analyzed the impact of the five scales on classification accuracy. In addition, the effectiveness and redundancy of features obtained by the different scale parcellations were compared. Finally, traditional statistical significance (*P* value) was verified as a feature selection criterion. The results showed that the feature effectiveness of different scales was similar; in other words, parcellation with more regions did not provide more effective discriminative features. Nevertheless, parcellation with more regions did provide a greater quantity of discriminative features, which led to an improvement in the accuracy of the classification. However, due to the close distance between brain regions, the redundancy of parcellation with more regions was also greater. The traditional *P* value feature selection strategy is feasible with different scales, but our analysis showed that the traditional *P* < 0.05 threshold was too strict for feature selection. This study provides an important reference for the selection of network scales when applying topological properties of brain networks to machine learning methods.

## 1. Introduction

Machine learning and pattern recognition methods have been widely used in functional magnetic resonance (fMRI) data analysis in studies of brain thinking and cognitive state (for review, see [1]). The classification features selected for fMRI analysis are mostly direct features of the blood oxygen level-dependent (BOLD) signal, including peak, peak time, and slope (for review, see [2]), regardless of whether the analysis involves task-state fMRI [3, 4] or resting-state (rs) fMRI [5].

In recent years, with the development of functional brain network research, more and more researchers have found that the rich topological information of functional networks can be used as biological markers for various neuropsychiatric diseases [6–9]. The extracted network topological features are widely used in the construction of classification models to assist in the diagnosis of brain diseases (for review, see [10]).

The topological features selected have usually included global properties [11], local properties [12], community structures [13], and connections [14]. In recent years, researchers have proposed new methods for network feature analysis, which have been applied in brain disease machine learning research, such as hypergraph [15], high-order network [16], minimum spanning tree [17], and frequent subgraphs [18] methods. Brain network topological features provide a new perspective for combined research using fMRI and machine learning.

At present, this field is still in the exploration stage, and many methodological issues still need to be resolved. One of the important issues is how to make a reasonable parcellation selection to define network nodes. Previous studies have found that different network node scales (related to different brain network parcellation templates) significantly impact the structure of the constructed network and its topological properties [19, 20], such as the network's small-world properties [19, 21], local properties [19–21], functional connection strength [19], and network connectivity [19].

In addition, the impact of network node scale on the network is reflected in the classification features by network topology properties. When the discriminative features extracted from networks with a different number of nodes are applied to machine learning classifiers, they influence the classification accuracy. In a previous study, Jing et al. [22] used two scales of the Anatomical Automatic Labeling (AAL) atlas, AAL-90 (90 nodes), and AAL-1024 (1024 nodes) to study the identification of major depressive disorder (MDD) patients, and they found that the recognition performance of the AAL-1024 parcellation was better than that of the traditional AAL-90 parcellation. In another study, Ota et al. [23] used AAL (90 nodes) parcellation and LPBA40 (54 nodes) parcellation to study the impact of parcellation and discriminative feature selection on the prediction of Alzheimer's disease (AD), and they found that the classification accuracy was low when there was a small number of nodes. Mesrob et al. [24] used AAL (90 nodes) parcellation and 487ROI (487 nodes) parcellation for the identification of patients with AD/mild cognitive impairment. Abnormal properties were identified as classification features, and the accuracy of the classification features was higher for a large network than for a small network.

Previous studies have consistently verified that the network scale affects classification accuracy; the accuracy of parcellation with more regions is higher than that associated with fewer regions. However, a potential problem in the previous studies was that the number of parcellations used was few (only two mostly), which did not allow for much comparison. In addition, previous studies only analyzed the final classification accuracy and ignored the actual performance of the selected features.

Furthermore, in terms of the feature selection, many previous studies have used the statistical analysis *P* value as the feature selection criterion. A review was conducted of 76 recent studies on machine learning related to brain networks (Supplemental Material [Supplementary-material supplementary-material-1]). Some of these studies were cited in a 2016 review of the literature on machine learning and brain networks [25] and a 2016 review of the literature on depression and machine learning [26]. Additional studies published in the past three years (2016–2018) were included. The review showed that there were 31 (40.78%) studies that selected the *P* value or a combination of the *P* value and other metrics as the feature selection strategy. This result showed that the *P* value is one of the most common feature selection strategies in this field. However, there is a potential risk in that the threshold *P* value was always arbitrarily set as 0.05 or 0.01, ignoring the possible contribution of additional features to the classification.

At present, we still do not know precisely how network scale affects the performance of classification features and classification accuracy, and effectiveness of the feature selection strategy using *P* values. In this context, this study applied five different node parcellations to construct and analyze the resting-state functional brain network of a control group and a disease group (involving patients with depression). The local topological properties with significant between-group differences were then extracted as classification features and applied to the classifiers. Finally, we analyzed the classification accuracy, feature performance, and feature selection strategy. To be precise, the study had the following four objectives: (1) to define five scale parcellations, comprising 90, 256, 497, 1003, and 1501 nodes; (2) to analyze the effects of these five parcellations on classification accuracy; (3) to analyze and compare the feature effectiveness and redundancy among the different scales; and (4) to investigate the feasibility and reasonable threshold regarding the *P* value feature selection strategy. This study provides an important reference for selecting the network scale for future applications of brain network topological properties to machine learning methods.

## 2. Materials and Methods

### 2.1. Proposed Framework

Data classification rs-fMRI methods usually include data preprocessing, construction of functional connectivity networks, feature selection, and classification. Specifically, the framework consists of the following steps:

1. Data acquisition and preprocessing.
2. Construction of functional connectivity networks.
  - Definition of parcellations. Application of *k*-means clustering to subdivide brain regions based on the AAL atlas.
  - Connection definition and threshold selection. Using the Pearson correlation approach, calculating the degree of correlation of the average time series of each region. Sparsity *S* is used for the threshold setting.
3. Feature selection and classification model construction (applying five node parcellations).
  - Calculation of basic local metrics: degree, betweenness centrality, and nodal efficiency for each node.
  - Use of the *P* value to select the discriminative features as classification features and constructing the classifier.
  - Use of cross-validation to test the constructed classifiers, obtaining the final classification results, and analysis of the effects of five scale parcellations on classification accuracy.
4. Feature effectiveness evaluation.
  - Evaluation of the effectiveness of the selected features, adopting the minimum redundancy-maximum relevance (mRMR) method.
  - Investigation of the feasibility and reasonable threshold regarding the *P* value feature selection strategy.

### 2.2. Subjects

In this study, 66 subjects were recruited, including 38 patients for the depression group and 28 age- and gender-matched healthy volunteers as a control group. All subjects were Han Chinese. Based on the Structured Clinical Interview for DSM-III-R Axis II Disorders (SCID-II) [27], no subjects in the control group had a history of mental or neurological disorders. All patients in the depression group were diagnosed with single-episode major depressive disorder, as defined by the Diagnostic and Statistical Manual of Mental Disorders Fourth Edition (DSM-IV) [28], at the Department of Mental Health, First Hospital of Shanxi Medical University, and all were medication naive. The severity of depression was assessed using the 24-item Hamilton Rating Scale for Depression (HRSD) [29] and Clinical Global Impression of Severity (CGI-S) [30]. Before scan, each subject provided written informed consent (the control group subjects provided consent by themselves and the depression group subjects provided consent with their family members). The demographic and clinical characteristics of the subjects are shown in Table 1.

### 2.3. Data Acquisition and Preprocessing

All subjects underwent rs-fMRI in a 3T magnetic resonance scanner (Siemens Trio 3-Tesla scanner, Siemens, Erlangen, Germany). Data collection and prepossessing were conducted at the First Hospital of Shanxi Medical University. All scans were performed by radiologists who were familiar with fMRI methods. During the scan, subjects were asked to relax and close their eyes but not fall asleep. Subjects were provided with soft earplugs and positioned carefully in the coil with comfortable support. Each scan consisted of 248 contiguous echo planar imaging (EPI) functional volumes (33 axial slices, repetition time [TR] = 2000 ms, echo time [TE] = 30 ms, thickness/skip = 4/0 mm, field of view [FOV] = 192 × 192 mm, matrix = 64 × 64 mm, and flip angle = 90°) and the first 10 volumes of time series data were discarded to allow for magnetization stabilization. See Supplemental [Supplementary-material supplementary-material-1] for detailed scanning parameters.

For the data preprocessing process, we followed the methods of one of our previous researches [31]. Data preprocessing was performed using Statistical Parametric Mapping (SPM8) software [32]. First, slice-timing correction and head motion correction were carried out. Three samples, exhibiting more than 3.0 mm of translation and 3.0° of rotation, were excluded, leaving a final dataset of 66 samples. The corrected images were then optimized using a 12-dimensional affine transformation and normalized to a voxel size of 3 × 3 × 3 mm in Montreal Neurological Institute (MNI) standard space [32]. Finally, linear detrending and bandpass filtering (0.01–0.10 Hz) were performed to reduce the effects of low-frequency drift and high-frequency physiological noise. Each regional mean time series was regressed against the mean cerebrospinal fluid and white matter signals as well as the six parameters from motion correction. The residuals of these regressions constituted a set of regional mean time series that were used for undirected graph analysis. Considering the debate regarding the validity of global signal regression in fMRI studies [33, 34], we did not perform global signal regression during preprocessing.

### 2.4. Definition of Parcellations

To achieve many different segmentation parcellations, we adopted the *k*-means clustering algorithm. Additionally, to avoid the influence of randomness in the initial seed set on the segmentation parcellation, we used a random seed set method. Thus, a random seed voxels method, based on *k*-means clustering, was used to subdivide brain regions based on the AAL atlas. We dynamically adjusted the location of the seed voxels to avoid a randomization impact on the node definition. The specific method was as follows. We tested 250, 500, 1000, and 1500 nodes, along with the definition of 90 nodes of the original AAL template, and a total of five parcellation templates were obtained. We calculated the total gray matter volume proportion, *V*, of each AAL node. We then determined the number of subregions as *k* = *VN* in each original region, such that the brain region (BR) was subdivided into *k* subregions under the *N* parcellation, where *N* is the expected number of nodes. Next, we designated *k* random seed voxels as *S* = *s*_1_, *s*_2_, *s*_3_,…, *s*_*k*_ in the BR. In turn, we then calculated the Euclidean distance between all residual voxels and the *s*_*i*_ seed voxels. Thereafter, the current voxel, *v*, was combined with the nearest seed voxel, *s*_*i*_, to define a new subregion, and the physical center of *v* and *s*_*i*_ was set as a new seed voxel. These steps were repeated until all voxels of the whole brain were divided. At this point, BR was divided into *k* subregions, and all brain regions after division showed the expected parcellation based on *N*.

It is worth noting that this method can only be applied to independent AAL brain regions when dividing brain regions. Voxels in adjacent brain regions of the AAL atlas remain separate at present, even if their Euclidean distance is small. In addition, the volume proportion, *V*, of the brain region is not always an integer, in which case, the values are rounded. This produces a slight deviation between the actual number of nodes and the expected number of nodes, *N*.

We tested *N* values of 250, 500, 1000, and 1500, comprising 256, 497, 1003, and 1501 regions, respectively. Including the original AAL template, this study thus defined five parcellations, designated AAL90, Parc256, Parc497, Parc1003, and Parc1501. The prefix AAL denoted the original AAL atlas. The prefix Parc denotes templates, which were determined using the algorithm described above (For an illustration of the five parcellations, see Supplemental Material [Supplementary-material supplementary-material-1]. For the Nii files, see Supplemental Material Digital Files [Supplementary-material supplementary-material-1]. For an illustration of the parcellation definitions, see Supplemental Material [Supplementary-material supplementary-material-1]).

### 2.5. Connection Definition and Threshold Selection

For the connection definition and threshold selection, we followed the methods of one of our previous researches [31]. The Pearson correlation coefficient was used to define the network edge in this study. First, we calculated the mean time courses of each node and then performed multiple linear regression to remove the pseudodifferences caused by head movement. The residuals were used to compute the partial correlation, producing an *N* × *N* correlation matrix, where *N* represents the number of nodes in a given parcellation. According to the predetermined threshold, the correlation matrix was converted into a binary matrix (For the mathematical definition of the Pearson correlation coefficient, see Supplemental Material [Supplementary-material supplementary-material-1]).

Sparsity, *S*, which is the ratio of the number of real existing edges to the maximum possible number of existing edges [35], was used as the threshold setting. This method has been widely adopted in similar studies [36–39]. To ensure the comparability of results between the parcellations, the threshold space *S*∈(8%, 32%) of the AAL90 parcellation was used as the standard for all five parcellations, and the brain functional networks of all subjects were constructed with an *S* step size of 5% within the threshold space (For details of the threshold selection criteria, see Supplementary Material [Supplementary-material supplementary-material-1]).

To characterize the integrity properties of a metric in the complete sparsity space, we calculated the area under the curve (AUC) for each metric. AUC provides a method to assess the total change in the network node properties under different degrees of sparseness. This method has been applied in previous research, which showed that it represents a very sensitive method to assess changes in the topological properties of a brain network [21].

### 2.6. Network Metrics and Statistical Analysis

Network metrics can characterize the topological properties of a network [40]. We chose three basic local metrics: degree, betweenness centrality, and nodal efficiency (For the mathematical definitions and interpretation of these network metrics, see Supplementary Material [Supplementary-material supplementary-material-1]).

The nonparametric Kolmogorov–Smirnov test was to determine whether there were significant differences in the network metrics for each brain region between the depression and normal groups [41]. Thereafter, the Benjamini & Hochberg false-discovery rate (FDR) method (*q* = 0.05) was used. This FDR method, which retains strong control over type 1 errors in the context of multiple comparisons, is considered appropriate to correct the comparative results of small samples [42].

### 2.7. Feature Selection and Classification

The process of feature selection and cross-validation included the following steps: (1) calculating three local properties (degree, betweenness centrality, and nodal efficiency) at each scale; (2) performing the Kolmogorov–Smirnov test for each property at each scale; (3) selecting the properties with significant between-group differences as discriminative features to construct feature subsets (*P* < 0.05, FDR corrected) at each scale separately; and (4) conducting cross-validation (10-fold, 100 times) at each scale separately.

The support vector machine (SVM) method was used to construct the classifiers, which has been frequently used in previous research [43–46]. In particular, the SVM method exhibits a good classification effect for small sample data [47]. The LIBSVM Toolkit (http://www.csie.ntu.edu.tw/∼cjlin/libsvm) was used, and the SVM parameter settings were as follows: kernel function = linear; stopping criteria = 1 × 10^−3^; regression precision = 0.1; bias = 0; and degree = 3.

Selecting the kernel function in the classification is the key. Linear kernel function was used for classification, and 10-fold cross-validation [48] was used to evaluate the classifier performance (it was repeated 100 times to obtain more accurate results). For cross-validation, the data were randomly divided into two sets: a training set and a test set. The training set was used to estimate the model parameters, and the test set was used to evaluate the model. More precisely, the subjects were randomly divided into ten equal parts, of which nine represented the training set, and the remaining one represented the test set.

### 2.8. Classification Feature Evaluation

To evaluate the effectiveness of the selected features, we adopted the mRMR method [49]. Using this method, mutual information was used to evaluate the degree of correlation between the features and groups (i.e., depression and control groups). This method can also assess the similarity between selected features. Metric mutual information difference (MID) represents the difference between the maximum correlation value and minimum redundancy value; in other words, the information gap. Metric redundancy (*R*) represents the dependency relationship between the discriminative features. The correlation between each of the discriminative features should be minimal; that is, the principle of minimum redundancy. For the mathematical definitions of these metrics, see Table 2. For details of mRMR, see Supplemental Material [Supplementary-material supplementary-material-1]. We used the mRMR Toolkit (http://home.penglab.com/proj/mRMR/) to calculate the MID and *R* values.

## 3. Results

### 3.1. Feature Selection and Classification Based on *P* < 0.05

We constructed functional networks with five scales and calculated three local topological properties (degree, betweenness centrality, and nodal efficiency). The AUC method was performed to unify local properties among different sparsities. The total number of features were 270 (AAL90), 768 (Parc256), 1491 (Parc497), 3009 (Parc1003), and 4503 (Parc1501). The *P* value was used as the feature selection criterion, and local topological properties with significant between-group differences (*P* < 0.05, FDR corrected) were used as discriminative features. The results showed that as the number of network nodes increased, the number of discriminative features for each local property increased; the classification accuracy of the classifier also rose (Table 3).

**Figure 1:**
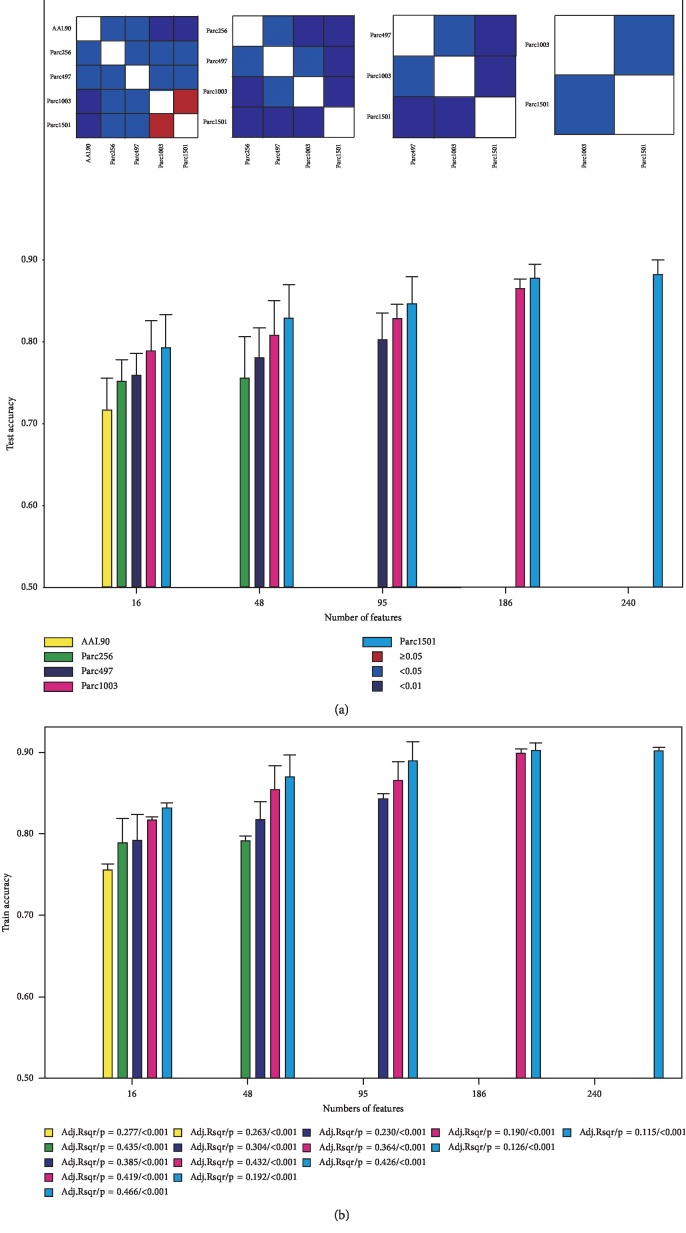
Mean accuracy associated with the same number of discriminative features at five scales. The color map shows the statistical significance between each scale pair.

**Figure 2:**
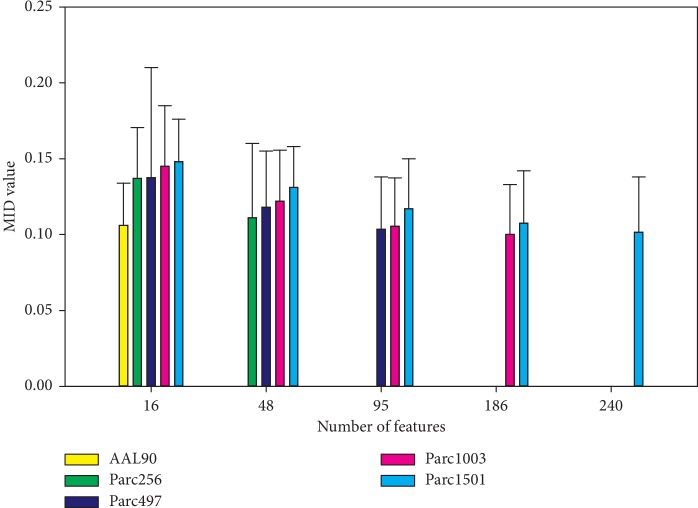
MID values associated with same number of discriminative features at five scales.

To eliminate the influence of the number of features, the same number of features was selected among different scales, and the classifiers were reconstructed (including 10-fold cross-validation with 100 repeats). Specific classification steps were descripted in Section 2.7. The results showed that the higher the number of nodes, the higher the classification accuracy (Figure 1(a)). Additionally, the classification feature performance of a large network was better than that of a small network with the same number of features (Figure 2). Here, in Figures 1 and 2, the number of features, 16, 48, 95, 186, 240 in *x* axis, was defined by the number of discriminative features obtained at each node scale (corresponding with AAL90, Parc256, Parc497, Parc1003, and Parc1501). The histogram with bar with an abscissa of 16 indicates the classification accuracy of the selected top 16 discriminative features sorted by the *P* value from lowest to highest at each scale. It should be noted that the histogram with bar with an abscissa of 48 represents the accuracy of 48 discriminative features at each scale except AAL90. Because when the node scale is 90, the number of discriminative features is not enough (only 16). The classification accuracy of discriminative features obtained from AAL90 is not listed at the abscissa of 48. Other numbers of features were similar.

Furthermore, in order to verify whether there was overfitting problem at each node scale, we calculated the training accuracy and performed the regression analysis between training accuracy and test accuracy at each node scale (Figure 1(b)). Specifically, similar to the test accuracy, 10-fold cross-validation with 100 times was performed to calculate training accuracy, whose arithmetic mean was used as the final training accuracy. The results show that the difference between the training accuracy and the corresponding test accuracy at each feature scale is not very large and is about 2%–4%. Moreover, regression analysis was performed between the training accuracy and test accuracy at each node scale. The results showed that at each node scale, regardless of which feature numbers, the training accuracy was positively correlated with the test accuracy, that is, the higher the training accuracy, the higher the test accuracy. It can be seen that the classification of different feature numbers was not affected by overfitting significantly. A more detailed discussion about overfitting problem can be seen in Section 4.5.

### 3.2. Effectiveness Analysis of all Features

**Figure 3:**
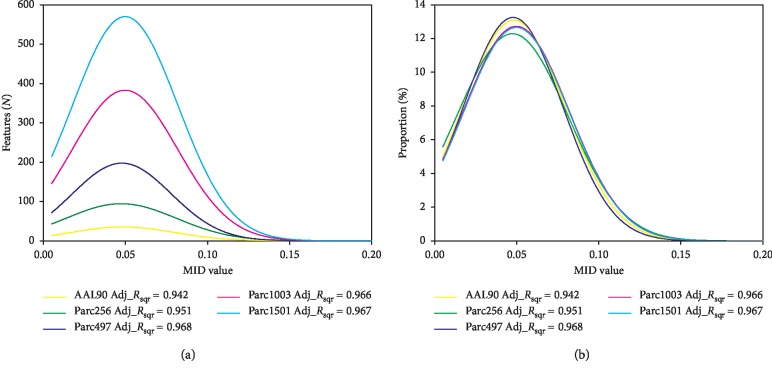
Frequency and proportion distribution of MID values for all features at five scales. (a) Frequency distribution. (b) Proportional distribution. Adj_*R*_sqr_, adjusted *R*^2^.

To illustrate the classification performance of all features for the five scales, we calculated the effectiveness of each feature and then analyzed the proportion and frequency distributions. The results showed that the distributions of all five scales were Gaussian (Figure 3). The larger the network, the greater the number of features (Figure 3(a)). In addition, the frequency distribution showed that the feature-fit curves of the five scales overlapped (Figure 3(b)), suggesting that an increase or decrease in the number of network nodes did not directly affect the effectiveness of the features.

**Figure 4:**
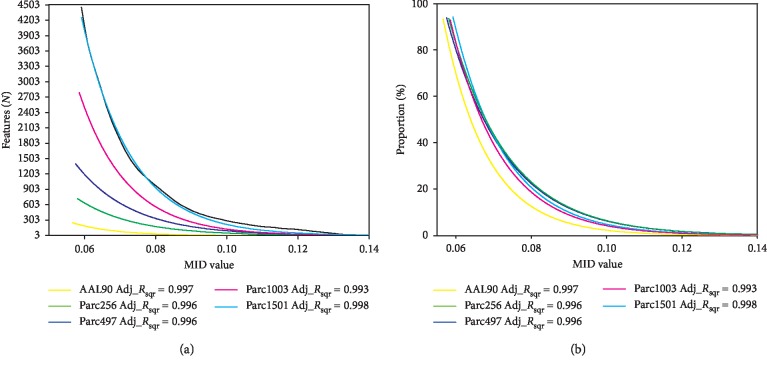
Frequency and proportion distribution of mean MID values as the features increased at five scales. (a) Frequency distribution. (b) Proportion distribution. Features were sorted by *P* value (from small to large) and the step size was three. Adj_*R*_sqr_, adjusted *R*^2^.

Previous research used *P* values as feature selection criteria [42]. To study the change in effectiveness for all features under this standard, we sorted the features by *P* value (small to large), performed feature selection (taking three features as the step size), and then calculated the mean MID value for all the feature subset (Figure 4). The results showed that the MID values of the five scales were consistent and conformed to the exponential decay function (Figure 4(a)). Proportion analysis showed that the distribution functions of the five scales were very close (Figure 4(b)).

### 3.3. *P* Value: the Feature Selection Criterion

**Figure 5:**
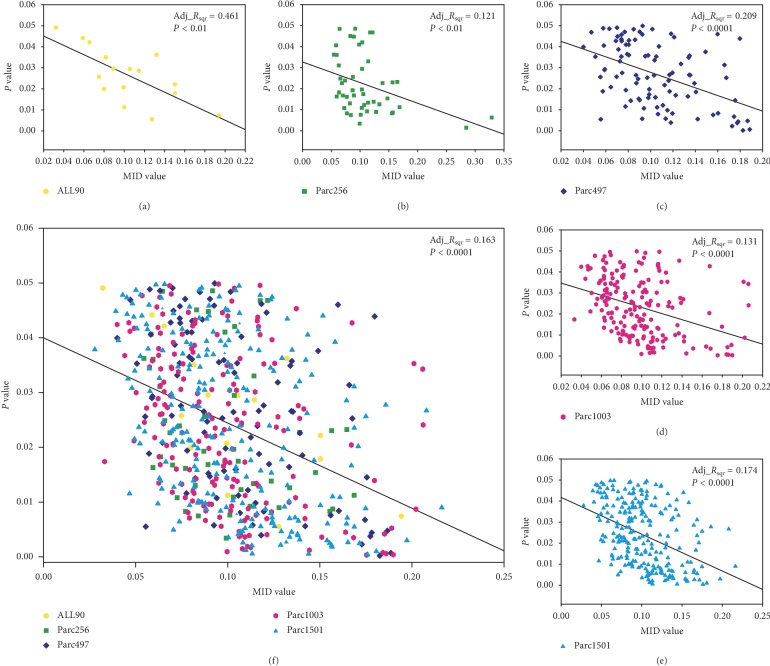
Correlation analysis between *P* values and MID values. (a–e) Correlation data for each of the five scales. *F* Correlation of all discriminative features. Adj_*R*_sqr_, adjusted *R*^2^.

**Figure 6:**
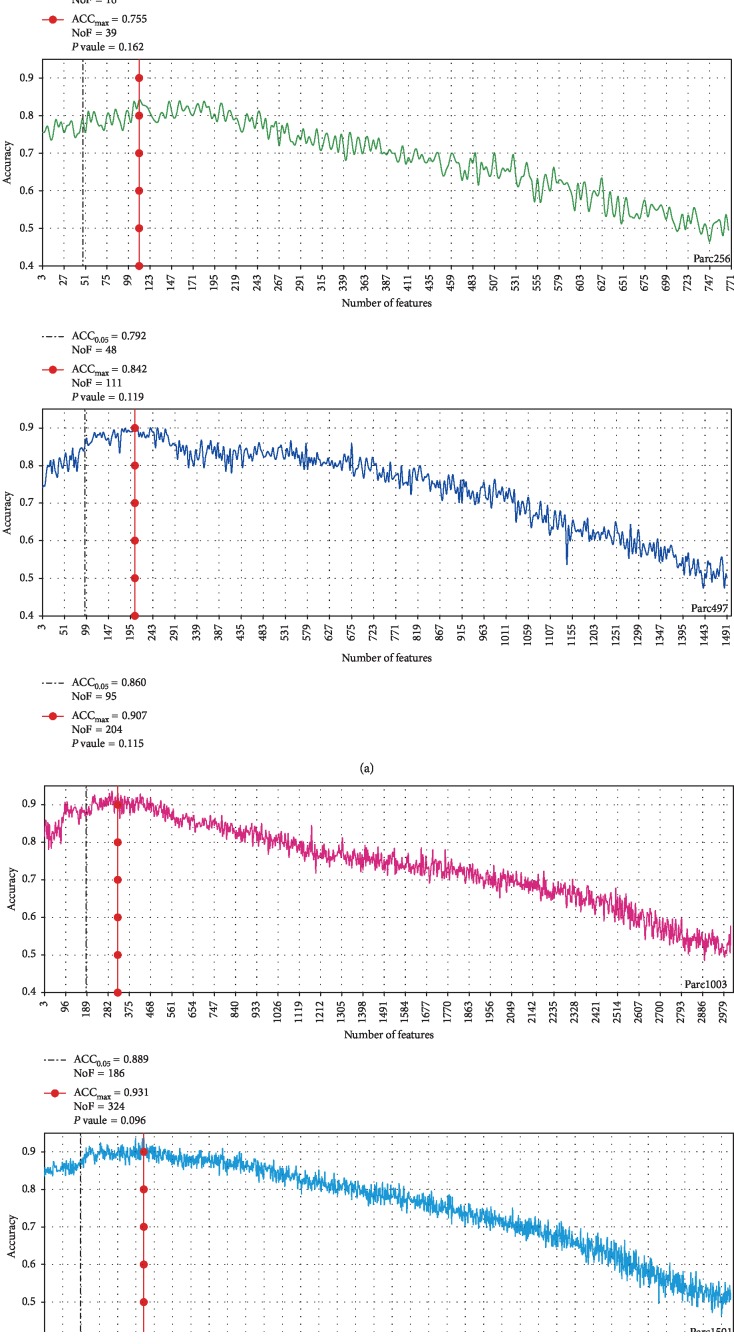
Classification accuracy with an increasing number of features at five scales. The black dotted line indicates the position where *P* ≈ 0.05; the red solid line with a red circle indicates the peak value. ACC_0.05_, classification accuracy at *P* ≈ 0.05; NoF, number of features; ACC_max_, peak classification accuracy.

To verify the performance of the *P* value as the feature selection criterion among the five parcellations, we analyzed the correlation between the *P* value and the MID value for each scale by linear regression. The results showed that there was a significant negative correlation between these parameters (*P* < 0.01), regardless of the scale (Figure 5). It should be noted that all the significant correlation were weak (the adjusted *R* square values were around 0.16) except for AAL90 parcellation (the adjusted *R* square values was 0.461). In addition, to identify the optimal feature subset, we analyzed the classification performance of all the features. All the features were sorted by *P* value and feature subsets were then selected in turn using three as the step size. The feature subsets were then used to train the classification model in sequence. In consideration of computational consumption issues, classification with each feature subset was repeated five times.  Figure 6 shows the change in the mean classification accuracy with an increasing number of features at the five scales. The results showed that all scales exhibited a similar trend. In the beginning, the accuracy of classification increased as the number of features increased. Then, as the effectiveness of the added features decreases, the classification accuracy also gradually decreases. In particular, when all features of each scale were selected as classifier features, the accuracy was approximately 50%. This means that the classifier degenerated into random classification.

In addition, the results of the five scales showed that accuracy was not optimal when the *P* value was set at 0.05. The classification results of the five scales all showed the same phenomenon, which suggested that 0.05 was not the optimal *P* value threshold when filtering classification features. When the *P* value was 0.05, the classification accuracy rate was still increasing, regardless of the scale. The number of features/approximate *P* values corresponding to the highest accuracy for the five scales were as follows: 39/0.162, 111/0.119, 204/0.115, 324/0.096, and 654/0.126. This result implies that the feature selection criterion of *P* < 0.05 is too strict to ensure the highest accuracy.

### 3.4. Feature Redundancy Analysis

**Figure 7:**
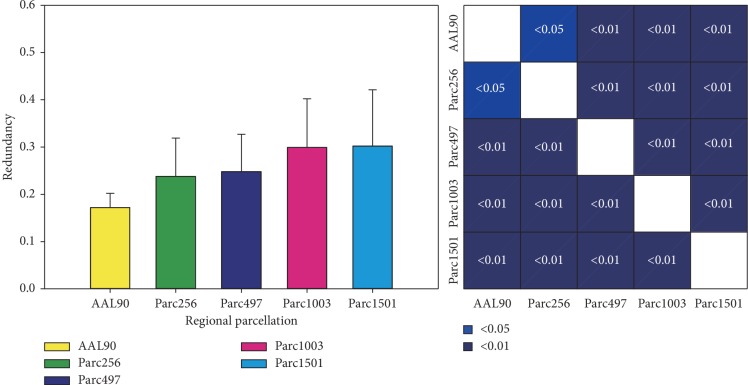
Redundancy between features at five scales. The color map shows the statistical significance of redundancy between each scale pair. *R*, redundancy. Error bars show the standard deviation.

When evaluating feature performance, besides the effectiveness of selected features, the similarity between features should also be evaluated; in other words, feature redundancy, *R* (the mathematical definition of which is shown in Table 2). The redundancy between each feature pair at all five scales was calculated separately (considering the computational consumption, only the feature subset associated with the *P* < 0.05 criterion was selected). The results showed that the redundancy between the discriminative features gradually increased as the number of nodes increased (Figure 7). This suggested that although parcellations with more regions provided more discriminative features, the redundancy between these features was also strong; that is, the similarity between features was high.

**Figure 8:**
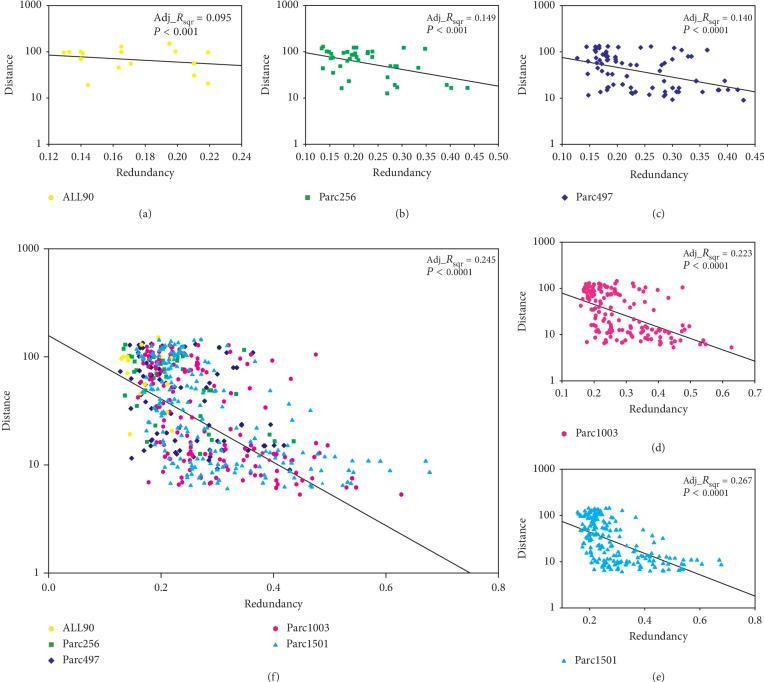
Correlation analysis between the anatomical distance of brain regions and redundancy. (a–e) Correlation data for each of the five scales. (*F*) Correlation for all five scales. *R*, redundancy. Adj_*R*_sqr_, adjusted *R*^2^.

We speculated that the increased redundancy between discriminative features may have been caused by the shorter anatomical distance between brain regions. To verify this conjecture, we performed a correlation analysis between the redundancy and the Euclidean distance between the corresponding brain region pairs (Figure 8), which identified a significant negative correlation. This suggested that a parcellation with more regions, which would have a shorter mean anatomical distance between regions, would have increased redundancy between the discriminative features of corresponding regions. This implied that the network topological properties of regions with a short anatomical distance were similar.

## 4. Discussion

The brain network method has provided novel viewpoints and ideas for studying the human brain from the perspective of complex networks. The definition of nodes is an extremely important issue when using this method. Different node definitions will result in different network node scales. Previous studies have shown that network node scale impacts network topological structure and classification accuracy [22–24]. Our study focused on feature performance analysis and feature selection criteria at different scales. The results showed that the performances of all the classification features associated with different network scales were similar. Parcellation with more regions provided a larger number of discriminative features. However, due to the shorter distance between brain regions, the redundancy of features in the large networks was also higher. At the same time, use of traditional feature selection criterion, the *P* value, is feasible at different scales, but the threshold of 0.05 is too strict.

### 4.1. Feature Selection Based on *P* < 0.05

The *P* value, as the most commonly used feature selection criterion, has been widely used in machine learning research based on image data [1], including machine learning brain network studies (for review, see [10]). Our study applied this common method and adopted 0.05 as a threshold to carry out feature selection and classification. The results showed that the greater the number of network nodes, the higher the number of discriminative features. This indicates that an increase in the network scale affected the number of discriminative features. This result is consistent with the results of similar studies [22, 50]. In addition, classification performance analysis showed that a large network significantly improved the accuracy compared with a small network (Table 3). This result is also consistent with the results of previous studies [22–24].

Based on the *P* < 0.05 criterion, different parcellations generated a different number of features. To remove the influence of the number of features on classification accuracy, we selected the same number of discriminative features from different scales and applied them to the classifier. The results were consistent with the earlier results (Figure 1). This indicated that the improvement in accuracy was not only caused by an increase in the number of features. Furthermore, after evaluating the performance of the selected discriminative features, it was found that the larger the network, the better the performance, which could lead to the improvement of classification accuracy (Figure 2). This implies that, when we want to control the quantity of features, the features provided by parcellation with more regions are more effective and more beneficial regarding classification accuracy improvement.

### 4.2. Analysis of All Features Based on Five Parcellations

The *P* value feature selection strategy only focuses on features with significant between-group differences, but it ignores other features; this approach is not, therefore, appropriate for a comparative study of features. Therefore, we analyzed all features obtained based on the five scales. Obviously, the larger networks had a greater number of local features. Classification performance analysis showed that the MID value distributions of the five scales were all consistently Gaussian (Figure 3(a)). Moreover, their proportional distribution functions were very similar (Figure 3(b)).

Furthermore, features were sorted by *P* value (from small to large), feature subsets were generated using three as the step size, and the mean MID value of each feature subset was calculated. The results showed that the mean MID values were higher for large networks compared to small networks when the number of features remained the same. This conclusion was consistent with the conclusion obtained using a feature subset associated with the *P* < 0.05 criterion. The trends in MID values for the five scales were consistent (as they were all in line with exponential decay) (Figure 4(a)); similar results were obtained in the proportion analysis (Figure 4(b)). Therefore, we can conclude that network scale did not affect the classification performance of the generated features, and that the effectiveness of the features obtained using different scales was similar.

### 4.3. Is *P* < 0.05 the Best Choice?

Two methods were used for feature evaluation: *P* values and MID values. Correlation analysis was performed between these two parameters, which showed that there were significant correlations, regardless of the scale. This also indicated that discriminative feature selection using *P* values was effective, and the effectiveness was not affected by differences of network scale. However, it is worth noting that regarding optimal feature subset selection, the threshold of 0.05 is arbitrary. Clearly, *P* < 0.05 can ensure the statistical significance of the selected features. However, this criterion appeared to be too strict for the selection of classification features. The full-feature classification results at the five scales showed that the change in the classification accuracy as the number of features (sorted by *P* value) increased involved two stages (Figure 6). First, there was a rising stage resulting from an increase in the number of effective discriminative features. Second, there was a declining stage caused by the reduction in MID values of newly added features. The results showed that the <0.05 criterion just fitted into the rising stage for all five scales. This indicated that this criterion is too strict as it leads to insufficient selection of features for the feature subset, thus reducing classification accuracy. The number of features/*P* values associated with the peak accuracy for each scale was as follows: 39/0.162 (AAL90), 111/0.119 (Parc256), 204/0.115 (Parc497), 324/0.096 (Parc1003), and 654/0.126 (Parc1501). Therefore, it could be worth considering setting the *P* value threshold for feature selection at 0.090–0.170 to improve the classification accuracy.

The construction of an optimal feature subset is a complex problem, involving the number of features, method of feature selection and effectiveness of the features [51]. The number of features plays an important role in the performance of the classifier [48, 51–53]. From a statistical point of view, a threshold of *P* < 0.05 ensured that the selected features were statistically significant. However, we also found that this criterion was too strict from the point of view of machine learning and led to fewer discriminative features being selected. It is therefore important to consider less strict criteria.

### 4.4. Feature Redundancy

Collectively, our results showed that large networks provide a larger number of discriminative features. We also need to pay attention to the redundancy between features, that is, the similarity between features. Similar features make similar contributions in classifiers [53]. If there are a large number of redundant features in the feature subset, the performance of the classifier is barely improved, even if these redundant features are discriminative features [54]. The redundancy analysis between the discriminative features of different scales showed that the redundancy was higher for a large network compared to a small network (considering the computational consumption, we only carried out the analysis for the feature subset associated with the *P* < 0.05 criterion). We believe that this was caused by the short anatomical distance between nodes for parcellations with more regions (Figure 8); this natural problem is unavoidable.

### 4.5. Issue of Overfitting

The overfitting problem refers to the fact that the machine learning model may perfectly predict the training set but fail to predict the new data very well if there are too many features, that is, the model is overfitted regarding the training data without considering its generalization ability. High-dimensional features can cause an overfitting problem.

Machine learning has been widely applied to extract information from fMRI data and predict pathology progression [55, 56]. From among the large number of machine learning methods, the classification method is particularly useful in pathology [57]. Moreover, research suggests that the SVM method is one of the most popular classification methods in machine learning involving neuroimaging data [58]. The SVM algorithm allows the classification of individual samples into distinct groups based on data in high-dimensional space according to the structural risk minimization principle [56]. In addition, SVM computational complexity is determined by the number of samples rather than the number of features, which is beneficial in high-dimensional settings. Regarding the problem of the data dimension exceeding the number of samples, SVM can always find the linear decision boundary to completely separate the data (via linear kernel) [56]. Therefore, SVM does not require many training samples to avoid overfitting [59]. Accordingly, SVM has attracted attention from neuroimaging researchers and has been used to extract meaningful information from high-dimensional fMRI data [56, 60].

The penalty parameter, *C*, using the training data optimization algorithm is included in the SVM model. The selection of appropriate parameters allows corresponding control over the overfitting phenomenon. *C* is used to control the tradeoff between model complexity and approximation error. If *C* is too large, the data fit and the complexity of the learning machine will be too high. Avoiding overfitting is a necessary process when designing a classifier. Conversely, if *C* is too small, the penalty for empirical error will be smaller, and the machine's learning complexity and data fit will be low. When there is overfitting or underfitting, the classifier will have a poor generalization ability and poor classification performance [61]. Thus, it is very important to choose a suitable *C* value, which would avoid the overfitting problem to a certain extent.

Furthermore, we assessed the issue of overfitting in our results. A linear kernel function was used for classification, and 10-fold cross-validation was used to evaluate the classification performance. Analysis in Section 3.1 showed that our results were not significantly affected by overfitting problem at all five parcellations in which training set and test set were set a ratio of 9 : 1. In order to avoid the effect of different proportion of the training and test set, in this section, we adopted a different ratio setting, which is 7 : 3. Specifically, we randomly divided the dataset into a training dataset and a test dataset in a 7 : 3 ratio, where 70% of the data were used as training data, and the remainder was not involved in model training. Furthermore, the training dataset was subjected to 10-fold cross-validation. That is to say, the training dataset was randomly divided into ten equal parts, one of which was used as the validation set (Sn) and the remainder as the training set (S-n). S-n was then divided into two parts (training set B and test set *C*). Using training set B, classifiers were constructed by choosing different *C* values, and the *C* value that gave the highest classification accuracy regarding training set B was determined to be the best parameter. In this way, ten different models were built, and the accuracy of each model was calculated. Next, the test set *C* was subjected to each model separately, and the associated accuracy was calculated to prove the generalization ability of the model.

Because the feature dimension was higher than the number of samples, it is easier to determine the linear decision boundary to completely separate the data in the SVM training model process. This made the fitting difficulty relatively low. Moreover, the number of samples in this study was small, owing to the difficulty of data acquisition, so the learning curve of a model did not show a significant rise during multiple training processes and converged quickly. Therefore, the statistical of learning curves may not be obvious to illustrate the issues. Thus, we randomly selected a training set and test set at a 7 : 3 ratio 100 times and calculated the validation accuracy and test accuracy after 10-fold cross-validation every time for each scale. In addition, different SVM parameter settings led to different results. In the training model, the penalty parameter, *C*, was set in the [−5, 5] range with a step size of 1 because the classification result was the best in this range. Five parcellations were validated respectively. Correlation analysis was performed between validation accuracy and test accuracy (Supplemental Material [Supplementary-material supplementary-material-1]). The results showed that the validation accuracy of the three parcellations significantly positively correlated with test accuracy. The corresponding adjusted *R*^2^/*P* values were as follows: 0.271/<0.0001 (AAL90), 0.229/<0.0001 (Parc256), 0.194/<0.0001 (Parc497), 0.152/<0.0001 (Parc1003), and 0.091/0013 (Parc1501). This suggested that the overfitting effect was not significant.

However, it should be noted that the adjusted *R*^2^ values decreased and the *P* values increased gradually with the increase in the number of nodes. This phenomenon suggested that we cannot rule out the influence of overfitting on the results with further increases in the number of features. This issue should be focused on by researchers in the future. In the latest related studies, to improve classification accuracy, many different complex network construction and feature extraction methods have been applied [62–64]. However, if the SVM classifier is selected, the overfitting problem in the face of high-dimensional features must be taken into account. Therefore, if a higher number of brain nodes are defined, necessary feature dimension reduction methods, such as the principal component analysis method [65], need to be implemented to avoid the overfitting problem in future studies.

### 4.6. Limitations

Our study showed that large networks yield a larger number of discriminative features, thus improving the accuracy of classification. However, we cannot ignore the greater time consumption caused by constructing and analyzing large networks. In addition to network scale, time consumption is affected by a variety of factors such as algorithms and hardware configurations. Consequently, we need to establish a balance between classification accuracy and acceptable time consumption.

In addition, we concede that our choice of methods in the current study, including node definitions, classifiers, feature selection strategy, performance evaluation indicators, and so on, are not the best in the field. They have their own problems and there will be better methods to replace them. However, the focus of our research was not to find the optimal classification model construction method, but to prove that a change in the network scale impacts feature selection and classification performance. Thus, in the process of constructing classification models, we chose the most common methods, even though these methods were not the best choice. This makes our research more comparable to other related studies.

There are several other limitations that need to be considered. First, because of the difficulties of sample collection, the number of subjects in similar studies is often insufficient, especially when the subjects are patients. Second, only one segmentation parcellation template was used for each scale. This means that our results cannot completely exclude the impact of randomness caused by the seed set settings, although we did adopt a dynamic adjustment strategy. Third, only one feature selection strategy was adopted. To ensure the generalizability of the results, more feature selection criteria should be considered in follow-up studies. Fourth, only one classifier was used for classification, which prevented comparisons between classifiers. Fifth, although we evaluated feature redundancy, we did not further optimize the feature subset; this will be the focus of our follow-up study. Sixth, although there was no significant overfitting phenomenon in this study, we cannot rule out the influence of overfitting on the results when the number of features is further increased. Finally, we did not optimize the classification parameters. The application of a parameter optimization strategy would improve the classifier performance.

## 5. Conclusion

This study analyzed how network scale affected classification performance, and classification accuracy and effectiveness of the feature selection strategies using *P* values in terms of machine learning methods. When using *P* < 0.05 as the feature selection threshold, we found that the classification accuracy of a larger network was higher than that of a smaller network. This finding is consistent with the findings of similar studies. Further analysis showed that improvements in the accuracy of parcellation with more regions were not caused by the increase in the number of features alone. When the number of features remained the same, parcellation with more regions still provided better accuracy because of the more efficient combination of features. Therefore, when the number of features needs to be controlled, selecting a parcellation with more regions improves the classification performance.

After analyzing all of the features, we found that the effectiveness related to the different scales was quite similar. This implies that changes in scale did not affect feature effectiveness. In other words, parcellation with a greater number of regions did not lead to features that were more effective. However, parcellation with a greater number of regions provided a greater quantity of discriminative features, which led to an improvement in classification accuracy. Furthermore, the feature redundancy was higher for parcellation with more regions than parcellation with fewer regions because of the shorter anatomical distance between brain regions in the former.

Application of the traditional *P* value feature selection strategy was feasible with different network scales and was further verified in this study. It is worth noting that although the threshold of *P* < 0.05 fully guaranteed statistically significant between-group differences in the selected features, it was too strict from a machine learning point of view, leading to a lack of discriminative features. Thus, a more relaxed threshold should be considered.

## Figures and Tables

**Table 1:** Demographic and clinical characteristics of the participants.

	NC (*n* = 28)	MDD (*n* = 38)	*P* value
Age (years)	17–51	17–49	*t* = 0.76^a^
	(26.6 ± 9.35)	(28.4 ± 8.99)	*P* = 0.41^a^
Gender (male/female)	13/15	15/23	^*χ*2^ = 0.31^b^
			*P* = 0.55^b^
Handedness (*R*/*L*)	28/0	38/0	—
HAMD	NA	15–42 (22.8 ± 13.19)	—

Data are presented as range (mean ± SD) or frequency. HAMD, Hamilton depression rating scale; MDD, major depressive disorder; NA, not applicable; NC, normal controls; ^a^the *t* and *P* values were obtained by a two-sample two-tailed *t*-test; ^b^the *χ*^2^ and *P* values were obtained by a two-tailed Pearson's *χ*^2^-test.

**Table 2:** Maximum relevance-minimum redundancy terms.

Term	Abbreviation	Formula	Interpretation
Dependency	*D*	*D*=(1/|*S*|)∑_*i*∈*S*_*I*(*h*, *i*)	Discriminating features are highly correlated with groups, and the most relevant features of the selection and categorization variables are selected. That is, the feature can reflect information relating to the groups to the greatest extent

Redundancy	*R*	*R*=(1/|*S*|^2^)∑_*i*,*j*∈*S*_*I*(*i*, *j*)	Description of the dependency relationship between discriminative features. Minimal relevance between each discriminative feature is required; that is, the principle of minimum redundancy

Mutual information difference	MID	(*D* − *R*)	Difference between the maximum relevance and minimum redundancy, represented by the two optimization conditions

*I* refers to the mutual information value between two features (*i* and *j*). *D* refers to the mutual information value between the discriminative feature and the category (groups). *h* refers to the groups of the dataset. |*S*| refers to the number of feature sets. *R* refers to the redundancy between the features.

**Table 3:** Multinode scaling effects regarding the number of discriminative features and classifier performance.

Parcellation	AAL90	Parc256	Parc497	Parc1003	Parc1501
Features (*D*/NE/BC)	5/4/7	16/13/19	31/28/36	55/71/60	82/77/81
Total	16	48	95	186	240
Accuracy (%)	74.3	82.7	83.5	87.5	88.5
Sensitivity (%)	79.3	89.3	92.0	87.3	91.9
Specificity (%)	66.3	74.0	75.2	88.2	83.6

*D*, degree; NE, nodal efficiency; BC, betweenness centrality.

## Data Availability

The image data used to support the findings of this study were supplied by Taiyuan University of Technology and First Hospital of Shanxi Medical University under license and so cannot be made freely available. Requests for access to these data should be made to the corresponding author.

## Supplementary Materials

Supplementary Materials Supplemental Text S1. Image Acquisition. Supplemental Text S2. Mathematical Definition of Pearson Correlation Coefficient. Supplemental Text S3. Threshold Selection Criteria. Supplemental Text S4. Mathematical Definitions of Selected Network Metrics. Supplemental Text S5. Minimum Redundancy-Maximum Relevance Algorithm. Supplemental Figure S1. Illustration of Five Parcellations. Supplemental Figure S2. Illustration of Parcellation Definitions. Supplemental Figure S3. Correlation Analysis between Validation Accuracy and Test Accuracy. Supplemental Table S1. Comparison with Similar Researches. Supplemental Digital File S1. Nii Files of Five Parcellations.
