# Supplementary material for: Resting-State Functional Network Scale Effects and Statistical Significance-Based Feature Selection in Machine Learning Classification
Source: Comput Math Methods Med. 2019 Nov 4;2019:9108108. doi: 10.1155/2019/9108108 (PMC6875180; doi:10.1155/2019/9108108)
Supplement: Supplementary Materials — Supplemental Text S1. Image Acquisition. Supplemental Text S2. Mathematical Definition of Pearson Correlation Coefficient. Supplemental Text S3. Threshold Selection Criteria. Supplemental Text S4. Mathematical Definitions of Selected Network Metrics. Supplemental Text S5. Minimum Redundancy-Maximum Relevance Algorithm. Supplemental Figure S1. Illustration of Five Parcellations. Supplemental Figure S2. Illustration of Parcellation Definitions. Supplemental Figure S3. Correlation Analysis between Validation Accuracy and Test Accuracy. Supplemental Table S1. Comparison with Similar Researches. Supplemental Digital File S1. Nii Files of Five Parcellations. [file 9108108.f1.zip › 9108108.f1/Supplemental Material Table S1.docx]

**Supplemental Table S1. Comparison among Similar Researches**

| **Paper[year]** | **Image Type** | **Disease/Groups** | **Patients/N** | **Patients/Age** | **Control/N** | **Control/Age** | **Node Definition** | **Feature Selection Method** | **Features** | **Machine Learning Method** | **Validation** |
| --- | --- | --- | --- | --- | --- | --- | --- | --- | --- | --- | --- |
| Robinson EC et al. [2010] | sMRI | Age (Young / /Seniors) | / | 20-30,59-90 | / | / | AAL(90) | / | Edge FA | MLDA | ACC=87,SEN=90,SPE=88,Bayeserr.=0.87 |
| Hui Shen et al. [2010] | fMRI | Schizophrenia | / | 19-30 | / | / | AAL(90) | / | ROI Signal Correlations | C-Means | ACC=92,AUC=0.96 |
| C.-Y. Wee et al. [2011] | sMRI | MCI | 10 | 74,/-8.6 | 17 | 74±8.6 | AAL(90) | SVM-RFE | Local CC | SVM | ACC=89, AUC=0.93 |
| Nico U. F. Dosenbach et al.[2011] | fMRI | Age Groups | / | 7 to 30 | , | 7 to 30 | Learned(210) | T-test(p<0.001) | ROI Signal Correlations | SVM | ACC=91,SEN=90,SPE=92 |
| Jonas Richiardi et al. [2011] | fMRI | Resting/Watching Movie | / | 18-36 | / | / | AAL(90) | T-test (p <0.05) | Multi-band Time Series | Polythetic Decision Trees | ACC=97 |
| Zhang J et al. [2011] | fMRI | MDD | 30 | 18–60 | 63 | 16–81 | AAL(90) | / | Small-world, Efficiency, and Nodal Centrality | / | / |
| Jin C et al.[2011] | fMRI | MDD | 16 | / | 16 | / | AAL(90) | / | Degree , BC, Efficiency | / | / |
| Wei Cheng1 et al. [2012] | fMRI | ADHD | 101 | 12.08 ± 2.05 | 143 | 11.43± 1.86 | AAL(90) | 1.T-tests(p < 0.008)  2.BWAS | ROI Signal Partial , Full Correlations | SVM | ACC=76,SEN=63,SPE=85 |
| Hai Li et al.[2012] | DTI | ASD | 10 | 7-14 | 10 | 7-14 | JHU-DTI-MNI atlas(46) | SVM-RFE | Edge Connectivity | SVM | ACC=100 |
| Soltanian-Zadeh et al. [2012] | fMRI | Rest/Attention | 19 | 20-30 | 19 | 20-30 | AAL(90) | Recursive feature ranking , WL Kernel | ROI Signal Correlations | SVM | ACC=100 |
| Jonas Richiardi et al. [2012] | fMRI | MS | / | 29-45 | / | / | AAL(90) | / | ROI Signal Correlations | Ensemble of Functional Trees | ACC=83,SEN=82,SPE=86 |
| Danesh Shahnazian et al. [2012] | fMRI | Rest/Attention | / | 20-30 | / | / | Conditional Granger Causality Analysis(CGCA)(24) | / | Bi-variate Granger Causality Network | SVM | ACC=100 |
| D. S. Bassett et al. [2012] | fMRI | Schizophrenia | 29 | 41.3 ± 9.3 | 29 | 41.1 ± 10.6 | AAL(90) | / | Largest Connected Component Size Curve | SVM | ACC=75,SEN=85,SPE=64 |
| C.-Y. Wee et al. [2012] | sMRI,fMRI | MCI | 10 | 64-83 | 17 | 64-83 | AAL(90) | T-test,Linear,p(0.004-0.01) Polynomial,RBF kernels | Structural and Functional Nodal CC | Multi-kernel SVM | ACC=96, SEN=100, SPE=94, AUC=95, YDI=94, BAC=95, |
| Lord AHorn D et al.[2012] ] | fMRI | MDD | 22 | / | 22 | / | AAL(90) | mRMR | PI, LE/GE, LE, Degree and BC | SVM | ACC =99 |
| Guo H et al. [2012] | fMRI | MDD | 38 | 17–54 | 28 | 17–51 | AAL(90) | T-test ( p<0.05) | Small-world, Efficiency, and Nodal Centrality | SVM | ACC =79.27 |
| Bai F et al.[2012] | DTI | RGD,aMCI | 35 | / | 30 | / | AAL(90) | / | Topological Metrics | / | / |
| Z.-W. Peng, et al. [2013] | fMRI | AD,MCI | 25 | 65-83 | 25 | 65-83 | AAL(90) | T-test ,RFE(p<0.005) | ROI Signal Correlations | SVM | ACC=92, AUC=90 |
| Biao Jie er al.[2013] | fMRI | MCI | 12 | 75.0±8.0 | 25 | 72.9±7.9 | AAL(90) | Linear-kernel-based,Graph Kernel | Local CC, Local Connectivity and Global | SVM | ACC=92 |
| C.-Y. Wee et al. [2013] | fMRI | MCI | 29 | 68-80 | 30 | 68-80 | AAL(90) | Fused multiple graphical lasso | Temporal Sliding Window Region Activations | SVM | ACC=90, BAC=79, SEN=76, SPE=83, AUC=0.79 |
| M. R. Arbabshirani et al. [2013] | fMRI | Schizophrenia | 28 | 36.5 ± 11.3 | 28 | 39.7 ± 10.1 | ROI (358) | 1.T-test (p<0.05);  2. Correlation-based Feature Selection (CFS) | ROI Signal Correlations | SVM | ACC=96,SEN=100,SPE=92,PPV=92,NPV=100 |
| K.Caeyenberghs et al.[2013] | sMRI,fMRI | TBI | 16 | / | 17 | / | ROI(22) | T-tests(p<0.05) | Degree, Efficiency, Betweenness centrality | Modality | ACC=61,SEN=43,SPE=77 |
| Tao H et al. [2013] | fMRI | MDD | 15 | / | 37 | / | AAL(90) | / | / | / | / |
| Singh M et al.[2013] | sMRI | MDD | 93 | 18–60 | 151 | 18–60 | AAL(90) | / | Clustering ,Path Length | / | / |
| Qin J et al.[2013] | DTI | MDD | 29 | 22–53 | 30 | 23–54 | AAL(90) | / | Network Metrics | / | / |
| D. Zhu et al. [2014] | fMRI,sMRI | MCI, Schizophrenia | 10 | / | 18 | / | DICCCOL(358) | Edge T-test, CFS(p<0.005) | ROI Signal Correlations | SVM | ACC=96 |
| Fei, Fei et al. [2014] | fMRI | MCI | 12 | 75.0±8.0 | 25 | 72.9±7.9 | AAL(90) | DSM , WL kernel | GSpan Frequent Subnetworks | Graph-kernel | ACC=97,AUC=0.96 |
| D. Zhu et al. [2014] | fMRI,sMRI | MCI | 10 | (55-84), (66-84) | 18 | (55-84), (66-84) | DICCCOL(359) | CFS | ROI Signal Correlations | CFS | ACC=100, 96 |
| Ghanbariet al.[2014] | sMRI | ASD, Age | 24 | 7.8-18.3 | 59 | 7.8-18.3 | ROI(79) | Built-into NMF | Edge Probability | NMF, Graph Embeddings | / |
| Jie, Biao et al.[2014] | fMRI | MCI | 12 | 75.0±8.0 | 25 | 72.9±7.9 | AAL(90) | 1.T-test(P [0.05 0.08 0.10 0.12 0.15])  2.RFE:Graph Kernel (Denoted as RFE-GK) | Local CC | SVM | ACC=92,BAC=94,SEN=100,SPE=88,AUC=94 |
| Biao Jie et al.[2014] | fMRI | MCI | 12 | / | 25 | / | AAL(90) | M2TFS | Local CC | SVM | ACC=95,SEN=92,SPE=96,AUC=0.96 |
| Pariyadath Vani et al.[2014] | fMRI | Smoking Status | 21 | 28-50 | 21 | 28-50 | ICA ,Clustering(56) | SVM-RFE | Correlations within and between Subnetwork Regions | SVM | ACC=79, PPV=83 |
| L. Wang et al. [2014] | fMRI | MCI | 12 | 65-83 | 25 | 65-83 | AAL(90) | T-test(p<0.05),REF, gspan | Local CC | SVM | ACC=97, AUC=0.92 |
| KAMIYA Kouhei et al. [2015] | DTI | TLE | 44 | 21-45 | 14 | 21-45 | ROI(83) | Sparse Linear Regression | Local Network Measures | SVM | ACC=90, AUC=0.97 |
| Khazaee, Ali et al.[2015] | fMRI | AD | 20 | 74.85 ± 4.50 | 20 | 75.05 ± 4.92 | AAL(90) | Fischer Score(p < 0.005) | Local , global network measures | SVM | ACC=100 |
| Bo-yong Park et al.[2015] | sMRI,fMRI | BMI | 60 | 29 | 60 | 29 | AAL(90) | T-test,Permutation Testing, Region Prior,Functional-Structural Correlation | Edge Fiber Density , fMRI Mean Nodal Degrees | PLSR | MAE=15%, RMS=5.3 |
| John R et al. [2015] | fMRI | Age and Clincal Risk(Low, High) | / | 6-12months | / | / | ROI(230) | T-test , Linear Kernel (p < 2.2251e-308) | ROI Signal Correlations | SVM | ACC=81,75,SEN=78,81,SPE=84,69 |
| Qiu et al. [2015] | fMRI | Age | / | 22-79 | / | / | / | / | ROI Signal Partial Correlations | Linear Regression | r=0.59,RMS=12.9 |
| Sacchet et al. [2015] | sMRI | MDD | 14 | 18-55 | / | / | Desikan–Killiany Atlas(68) | T-test (p <0.05) | Global Network Measures (9) | SVM | ACC=72,SEN=71,SPE=72 |
| Brent C. Munsell et al. [2015] | fMRI | TBI and AD | / | 70 | / | / | AAL(90) | / | ROI Signal Partial Correlations | SVM | ACC=82,SEN=40,SPE=98,PPV=86,NPV=81 |
| C. J. Brown et al. [2015] | sMRI | Low Motor Function | 146 | 24-32 | 22 | 27-4 | AAL(90) | PCA | FA | SVM | ACC=72,SEN=77,SP=69 |
| Ota K et al.[2015] | fMRI | MCI | 40 | / | 23 | / | AAL(90) | SVM-RFE | GM Density | SVM | ACC=77 SEN=97 SPEC=50 |
| L. Zhan et al. [2015] | sMRI,MRI | NC/MCI/ AD | AD：39 MCI：112 NC：51 | ADNI-2 | / | ADNI-2 | ROI(113) | High Order SVD | Edge Tract Counts | SLR | ACC=71, SEN=68, SPE=72, AUC=0.76 |
| Chung AW et al.[2016] | sMRI | Low Motor Function | 55 | 28.28±2.25 weeks | 233 | 30.01±2.23 weeks | AAL(90) | Heat kernel methodology | Heat Flow Features | Gaussian Naive Bayes | ACC=82,SEN=75,SPE=83,fs=79 |
| C.-Y. Wee et al. [2016] | fMRI | ASD | 45 | 7--15 | 47 | 7--15 | AAL(90) | Lasso | ROI Signal Correlations | SVM | ACC=71, SEN=80, SPE=61, |
| J. Galvis et al. [2016] | DWI | Parkinson’s Disease | 58 | / | 131 | / | FreeSurfer(129) | T-test(p<0.005) | ROI Signal Correlations | SVM | BAC=60 |
| D. Gellerup. Et al.[2016] | fMRI | Parkinson’s Disease | 24 | 60 | 21 | 61.9 | Power (264) | mRMR | ROI Signal Correlations | Proximal SVM Ensemble | ACC=0.84, SEN=0.73, SPE=93 |
| Jin Y et al.[2016] | sMRI | ASD | 40 | six-month | 40 | six-month | Atlas(90,203,403) | T-test(p < 0.001), LASSO | Edge FA, MD and TC, Multiple Scales | SVM | ACC=76,SEN=72,SPE=79,AUC=0.8 |
| Mitra J et al. [2016] | sMRI | TBI | 179 | / | 215 | / | AAL(90) | NBS Edge T-test (p < 0.002), PCA | Edge FA | Random Forest | ACC=68,SEN=80,SPE=46,PPV=68,NPV=69 |
| Christopher D. Smyser et al. [2016] | fMRI | Age | 50 | 23-29weeks | 50 | the first week | ICBM462(55) | T-test Filter(p<0.005) | ROI Signal Correlations | SVM | ACC=84,SEN=90,SPE=78 |
| J. Kawahara et al. [2016] | sMRI | Motor,Cognitive Function,Age | / | / | / | / | ROI(90) | T-tests(p<0.05) | Edge Tract Counts | BrainNetCNN | r=0.31,0.19, 0.86,MAE=10,10.5, 2.3 |
| G. Ball et al. [2016] | fMRI | Preterm /term, age | 26 | 23 ,6-48,0 | 105 | 37,1-46,2 | ICA(71) | Boruta algorithm((p<0.05) | ROI signal covariances | SVM,RBF,Linear Regression | BAC=80,AUC=0.92, MSE=8.9 |
| C. J. Brown et al. [2016] | sMRI | Low Motor,  Cognitive Function | 139/155 | / | 13/29 | / | AAL(90) | / | Edge Tract Counts | Non-negative linear Regression | ACC=71,AOC=14.3,r=0.44 |
| Biao Jie et al.[2016] | fMRI | MCI | 12 | 75±8 | 25 | 72.9±7.9 | AAL(90) | T-test (p<0.05) | Clustering Coefficients | SVM | ACC=82.9 SEN=83.9 SPE=81.6 |
| Hao Guo et al.[2017] | fMRI | AD | 38 | / | 28 | / | AAL(90) | T-test (p<0.05) | / | SVM | ACC=98.1 SEN=98.9 SPE=96.6 |
| Ali Khazaee et al. [2017] | fMRI | MCI and AD | AD(34) MCI(89) | 72.5 | 45 | 75.9 | AAL(90) | FSFS and Fisher Algorithm | Local and Global Graph | SVM | ACC=93.3 |
| Seyed Hani Hojjati et al. [2017] | rs-fMRI | MCI and AD | MCI-C(18) MCI-NC(62) | 73.6 | / | / | AAL(90) | T-test(p < 0.05) Fisher Score, Chi-square Score, Gini Score, and Kruskal-Wallis Test(p<0.05) | / | SVM | ACC=91.4 SEN=83.2 SPE=95 |
| De Marco et al. [2017] | rs-fMRI | MCI | 50(31/19) | 73.86 | 50(25/25) | 69.54 | AAL(90) | LDA and QDA | Blood Oxygen Level Dependent-Connectivity | SVM | ACC=90 |
| Ioannis Gallos et al. [2017] | rs-fMRI | Schizophrenia | 72 | 38.16 | 74 | 35.86 | Clustering(40-65) | PCA /T-test(p < 0.01) | / | SVM | ACC=63.5 |
| Chen, Xiaobo et al. [2017] | rs-fMRI | MCI | 54 | / | 54 | / | AAL(90) | T-test ( p < 0.05) and LASSO regression | RMS for Each Signal | SVM | ACC=78,SEN=77.7,SPE=79.6 |
| Alam et al. [2017] | MRI | MCI and AD | AD(89) MCI(102) | AD 77.3 MCI 77.36 | 102 | 76.05 | AAL(90) | KPCA , LDA | / | SVM | ACC=86.5 |
| Xiaofeng Zhu et al. [2017] | MRI | MCI and AD | AD(51) MCI(99) | / | 51 | / | AAL(90) | Sparse Multi-task Learning Framework | / | SVM | ACC=92 |
| Gurevich Pavel et al. [2017] | MRI | MCI and AD | AD(70) | 69.8 | non-AD(88) | 71.4 | / | PCA | / | SVM | ACC=82 |
| Alam Saruar et al. [2017] | MRI | AD | AD 86 | 77.3 | 86 | 76.05 | PCA | PCA | Principal Coefficients | SVM | ACC=92.6,SEN= 97.7,SPE=95.6 |
| Yu Zhang et al.[ 2017] | rs-fMRI | MCI | 53 | / | 59 | / | AAL(90) | Sparse Regression | Weighted Clustering Coefficients | SVM | ACC=83.8,SEN= 79.3,SPE=88.1 |
| Zhang Y et al.[2017] | rs-fMRI | eMCI | 13F/16M | / | 17F/13M | / | AAL(90) | Sparse Learning | Weighted-graph Local Clustering Coefficients | SVM | ACC=88.1,SEN=86,SPE= 90 |
| Yousra Asim et al.[2017] | sMRI,MRI | AD | 100 | 55-90 | 100 | 55-90 | AAL(90),LPBA40 | PCA | GM | SVM | ACC=94 SEN=95 |
| Y Li et al.[2017] | fMRI | MCI | 28 | / | 33 | / | AAL(90) | T-test (p<0.05) | Clustering Coefficients | SVM | ACC=86.9 SEN=82.1 SPE=90.9 |
| Guo Hao et al.[2017] | fMRI | MDD | 38 | / | 28 | / | AAL(90) | FSFS | Subgraph | SVM | ACC=88.9 SEN=91.7 SPE=85.6 |
| Hojjati et al. [2018] | sMRI,rs-fMRI | MCI | 18 | 73.6 ± 15.7 | 62 | 73.0 ± 16.3 | AAL(90) +Dosenbach Atlas | T-test(p<0.05) | / | SVM | ACC=91.4/ACC=89 |
| Bouts, Mark et al. [2018] | fMRI | AD | 41 | / | 40 | / | AAL(90) | T-test(p<0.05) | / | Elastic Net Regression | ACC=0.90,SEN=0.89,SPE=0.90 |
| Zhao Feng et al [2018] | RS-fMRI | ASD | 47 | 10.7 ± 2.28 | 40 | 11.22 ± 2.34 | AAL(90) | LASSO | ROI Signal Correlations | SVM | ACC=81 |
| Yang P et al.[2018] | fMRI | MCI | 24 | / | 23 | / | AAL(90) | Group Lasso | ROI Signal Covariances | SVM | ACC=93.6 SEN=91.6 SPE=95 |
| Chen Zu et al.[2018] | fMRI | AD | 50 | / | 50 | / | AAL(90) | T-test ( p<0.05) | Three-element Vector | SVM-STM | ACC=87 SEN=87 SPE=87 |
| NianyinZeng et al.[2018] | fMRI | AD | 92 | / | 92 | / | AAL(90) | PCA | ROI Signal Covariances | SDPSO-SVM | ACC=81.2 |
| SI Dimitriadis et al.[2018] | fMRI | AD | 100 | 74.9±5.6 | 100 | 73.1±8.2 | AAL(90) | KNIME Plugin K-Surfer | / | RF | ACC=61.9 |
