# Supplementary material for: Resting-State Functional Network Scale Effects and Statistical Significance-Based Feature Selection in Machine Learning Classification
Source: Comput Math Methods Med. 2019 Nov 4;2019:9108108. doi: 10.1155/2019/9108108 (PMC6875180; doi:10.1155/2019/9108108)
Supplement: Supplementary Materials — Supplemental Text S1. Image Acquisition. Supplemental Text S2. Mathematical Definition of Pearson Correlation Coefficient. Supplemental Text S3. Threshold Selection Criteria. Supplemental Text S4. Mathematical Definitions of Selected Network Metrics. Supplemental Text S5. Minimum Redundancy-Maximum Relevance Algorithm. Supplemental Figure S1. Illustration of Five Parcellations. Supplemental Figure S2. Illustration of Parcellation Definitions. Supplemental Figure S3. Correlation Analysis between Validation Accuracy and Test Accuracy. Supplemental Table S1. Comparison with Similar Researches. Supplemental Digital File S1. Nii Files of Five Parcellations. [file 9108108.f1.zip › 9108108.f1/Supplemental Material Text S2.doc]

**Supplemental Text S2. Mathematical Definition of Pearson Correlation Coefficient**

We used Pearson correlation coefficient to calculate the correlation coefficient of average time series between any two nodes. The mathematical definitions follows below:

$$r=\frac{\sum_{i=1}^{n} (X_{i}-\overline{X})(Y_{i}-\overline{Y})}{\sqrt{\sum_{i=1}^{n} (X_{i}-\overline{X})^{2}}\sqrt{\sum_{i=1}^{n} (Y_{i}-\overline{Y})^{2}}}$$

X_i_ and Y_i_ respectively represent time series of voxel i and j. We then generated a N × N time series correlation matrix. Here, N is the number of node in the given parcellation.
