# Supplementary material for: Resting-State Functional Network Scale Effects and Statistical Significance-Based Feature Selection in Machine Learning Classification
Source: Comput Math Methods Med. 2019 Nov 4;2019:9108108. doi: 10.1155/2019/9108108 (PMC6875180; doi:10.1155/2019/9108108)
Supplement: Supplementary Materials — Supplemental Text S1. Image Acquisition. Supplemental Text S2. Mathematical Definition of Pearson Correlation Coefficient. Supplemental Text S3. Threshold Selection Criteria. Supplemental Text S4. Mathematical Definitions of Selected Network Metrics. Supplemental Text S5. Minimum Redundancy-Maximum Relevance Algorithm. Supplemental Figure S1. Illustration of Five Parcellations. Supplemental Figure S2. Illustration of Parcellation Definitions. Supplemental Figure S3. Correlation Analysis between Validation Accuracy and Test Accuracy. Supplemental Table S1. Comparison with Similar Researches. Supplemental Digital File S1. Nii Files of Five Parcellations. [file 9108108.f1.zip › 9108108.f1/Supplemental Material Text S4.docx]

**Supplemental Text S4. Mathematical Definitions of Network Metrics**

In the current research, regardless of the binary matrix A = [aij] or weight matrix W = [wij], there are many network metrics to be calculated, including three local properties. Given different definitions of some network indicators, such as aggregation coefficients, we use superscript B and W to represent binary and weighted networks respectively.

In particular, for the analysis of weighted networks, the weights are standardized using the average weights of all non-zero elements to achieve the same level of connectivity in different populations. All the formulas in this paper are based on graph G= (N, K). Where G is a graph or network, N is the number of nodes in it, and K is the number of connections.

The 3 node metrics are calculated in the current research: degree ki, center centrality bi, node efficiency ei.

Degree. Formally, the definition of node degree is:

or （1）

Among them, aij (wij) is the element of two value (weight) network A (W) （i，j）. Degree is a basic measure of connectivity between a node and other nodes in the network.

Node Efficiency. The node efficiency formula of node i is defined as follows: [1]:

（2）

Among them, dij represents the shortest path length between node i and node j. The shortest path length represents the shortest number of connections between two nodes in the binary matrix in all possible connection stiffness or the minimum sum of connections in the weight matrix. Node efficiency calculates the information transfer capability between the given node i and other nodes in the network.

Betweenness Centrality. The betweenness centrality of node i is calculated as follows: [2]:

（3）

Among them, is the number of shortest paths from node m to node n, andis the number of shortest paths from node m to node n passing through node i. The betweenness centrality shows the importance of a node transmitting information at other nodes.

**References：**

1. Achard S, Bullmore E (2007) Efficiency and cost of economical brain functional networks. PLoS Comput Biol 3: e17.
2. Freeman LC (1977) A Set of Measures of Centrality Based on Betweenness. Sociometry 40: 35-41.
