# Supplementary material for: Resting-State Functional Network Scale Effects and Statistical Significance-Based Feature Selection in Machine Learning Classification
Source: Comput Math Methods Med. 2019 Nov 4;2019:9108108. doi: 10.1155/2019/9108108 (PMC6875180; doi:10.1155/2019/9108108)
Supplement: Supplementary Materials — Supplemental Text S1. Image Acquisition. Supplemental Text S2. Mathematical Definition of Pearson Correlation Coefficient. Supplemental Text S3. Threshold Selection Criteria. Supplemental Text S4. Mathematical Definitions of Selected Network Metrics. Supplemental Text S5. Minimum Redundancy-Maximum Relevance Algorithm. Supplemental Figure S1. Illustration of Five Parcellations. Supplemental Figure S2. Illustration of Parcellation Definitions. Supplemental Figure S3. Correlation Analysis between Validation Accuracy and Test Accuracy. Supplemental Table S1. Comparison with Similar Researches. Supplemental Digital File S1. Nii Files of Five Parcellations. [file 9108108.f1.zip › 9108108.f1/Supplemental Material Text S5.docx]

**Supplemental Text S5. Minimum Redundancy - Maximum Relevance**

At the center of mRMR[[1](#_ENREF_1)], the mutual information I of two discrete historical variables x and y is expressed as a function of their individual marginal probabilities, p(x) and p(y), and of their joint probabilistic distribution p(x, y), as follows :For discrete variables, the mutual information I of two variables x and y is defined based on their joint probabilistic distribution p(x, y) and the respective marginal probabilities p(x) and p(y):

$I\left( x,y \right)=\sum_{i,j} p(x_{i},y_{i})\log\frac{p(x_{i},y_{i})}{p\left( x_{i} \right)p(y_{i})}$ (1)

This mutual information measure is used to evaluate the level of“similarity” between historical variables. The goal of minimum redundancy is to optimally select those variables that reveal a minimum of resemblance between them, thus making the selected set more representative or informative of the whole set.

Let S be the subset of variables to be selected. The minimum redundancy criterion can be described as

min$R_{i}=\frac{1}{{|S|}^{2}}\sum_{i,j\in S} I(i,j)$ (2)

where I(i, j) denotes the mutual information measure between the input’s historical variables, and |S| stands for the number of selected variables in S.

To measure the relevance of variables with regard to the target to before casted, the mutual information I(h, i) is used once more between the target h (future values of the series) and the input historical variables i. I(h, i) measures the strength of i relatively to the forecasting process. Accordingly, the maximum relevance criterion is obtained by maximizing the entire relevance of all variables in S:

$\mathrm{maxD}_{I}=\frac{1}{|S|}\sum_{i\in S} I(h,i)$ (3)

The MRMR variable set is obtained by optimizing both criteria in Eqs. (2) and (3). This in turn necessitates their combination into one single criterion. The two simplest combination schemes are expressed as follows:

max($D_{I}-R_{I}$) (4)

$\max\frac{D_{I}}{R_{I}}$ (5)

This paper uses Eqs. (4) for the implementation of mRMR, and records it as the MID (Mutual Information Difference).

**References：**

1. Peng, H., F. Long, and C. Ding, Feature Selection Based on Mutual Information: Criteria of Max-Dependency, Max-Relevance, and Min-Redundancy. 2005: IEEE Computer Society. 1226.
