# Supplementary material for: Resting-State Functional Network Scale Effects and Statistical Significance-Based Feature Selection in Machine Learning Classification
Source: Comput Math Methods Med. 2019 Nov 4;2019:9108108. doi: 10.1155/2019/9108108 (PMC6875180; doi:10.1155/2019/9108108)
Supplement: Supplementary Materials — Supplemental Text S1. Image Acquisition. Supplemental Text S2. Mathematical Definition of Pearson Correlation Coefficient. Supplemental Text S3. Threshold Selection Criteria. Supplemental Text S4. Mathematical Definitions of Selected Network Metrics. Supplemental Text S5. Minimum Redundancy-Maximum Relevance Algorithm. Supplemental Figure S1. Illustration of Five Parcellations. Supplemental Figure S2. Illustration of Parcellation Definitions. Supplemental Figure S3. Correlation Analysis between Validation Accuracy and Test Accuracy. Supplemental Table S1. Comparison with Similar Researches. Supplemental Digital File S1. Nii Files of Five Parcellations. [file 9108108.f1.zip › 9108108.f1/Supplemental Material Text S1.doc]

**Supplemental Text S1. Image Acquisition**

All the subjects underwent resting state functional MRI scan using 3T MR equipment (Siemens Trio 3-Tesla scanner，Siemens, Erlangen, Germany). During the scan, subjects were instructed to relax with their eyes closed but not to fall asleep. Subjects were fitted with soft ear plugs and positioned carefully in the coil with comfortable support.

Each scan consisted of 248 contiguous EPI functional volumes (33 axial slices, repetition time (TR) = 2000 ms, echo time (TE) = 30 ms, thickness/skip = 4/0 mm, field of view (FOV) = 192×192 mm, matrix = 64×64 mm, flip angle = 90°) and the first ten volumes of time series were discarded for magnetization stabilization.
