# Supplementary material for: Resting-State Functional Network Scale Effects and Statistical Significance-Based Feature Selection in Machine Learning Classification
Source: Comput Math Methods Med. 2019 Nov 4;2019:9108108. doi: 10.1155/2019/9108108 (PMC6875180; doi:10.1155/2019/9108108)
Supplement: Supplementary Materials — Supplemental Text S1. Image Acquisition. Supplemental Text S2. Mathematical Definition of Pearson Correlation Coefficient. Supplemental Text S3. Threshold Selection Criteria. Supplemental Text S4. Mathematical Definitions of Selected Network Metrics. Supplemental Text S5. Minimum Redundancy-Maximum Relevance Algorithm. Supplemental Figure S1. Illustration of Five Parcellations. Supplemental Figure S2. Illustration of Parcellation Definitions. Supplemental Figure S3. Correlation Analysis between Validation Accuracy and Test Accuracy. Supplemental Table S1. Comparison with Similar Researches. Supplemental Digital File S1. Nii Files of Five Parcellations. [file 9108108.f1.zip › 9108108.f1/Supplemental Material Text S3.doc]

**Supplemental Text S3. Threshold Selection Criterions**

In the current study, sparsity, S, was used to set the threshold, which was defined as the ratio of the number of real existing edges divided by the number of maximum possible edges in the network. The selected threshold ensured that all networks from different groups had the same number of nodes and edges, which are the two golden rules for comparing complex networks [1] . Previous studies have reported that resting state functional brain networks exhibit the typical features of small-worldness (for review, see [2] ). To determine a data-specific small-world regime, each network was thresholded over a wide sparsity space with the following rules:

a) the average degree of all nodes is greater than 2×Lg (N), N = 90. N is the number of nodes in the network.

b) the small-worldness scalar σ> 1.1.

Determined by this procedure, threshold space is generated as S∈ (5%, 40%). There are five different parcellations in the current study, and the count of nodes are also unequal. Therefore the threshold value space is not consistent between the five parcellations. In order to make comparisons in uniform space, the threshold space above was used as the standard for all the five parccellations to structure the brain function network for all subjects and the interval was set as 5% throughout this study.

**References：**

[1]Bollob´as B. Random Graphs. Cambridge, UK: Cambridge Univ. Press; 1985.
[2]Bullmore ET, Bassett DS. Brain Graphs: Graphical Models of the Human Brain Connectome. Annu Rev Clin Psychol. 2011;7:113-140.
